# Animal models for SARS‐CoV‐2 infection and pathology

**DOI:** 10.1002/mco2.98

**Published:** 2021-11-16

**Authors:** Zhenfei Bi, Weiqi Hong, Jingyun Yang, Shuaiyao Lu, Xiaozhong Peng

**Affiliations:** ^1^ Laboratory of Aging Research and Cancer Drug Target State Key Laboratory of Biotherapy National Clinical Research Center for Geriatrics West China Hospital Sichuan University Chengdu Sichuan China; ^2^ National Kunming High‐level Biosafety Primate Research Center Institute of Medical Biology Chinese Academy of Medical Sciences and Peking Union Medical College Yunnan China

**Keywords:** animal models, COVID‐19, immunology, pathology, SARS‐CoV‐2, transmission

## Abstract

Severe acute respiratory syndrome coronavirus 2 (SARS‐CoV‐2) is the etiology of coronavirus disease 2019 (COVID‐19) pandemic. Current variants including *Alpha*, *Beta*, *Gamma*, *Delta*, and *Lambda* increase the capacity of infection and transmission of SARS‐CoV‐2, which might disable the in‐used therapies and vaccines. The COVID‐19 has now put an enormous strain on health care system all over the world. Therefore, the development of animal models that can capture characteristics and immune responses observed in COVID‐19 patients is urgently needed. Appropriate models could accelerate the testing of therapeutic drugs and vaccines against SARS‐CoV‐2. In this review, we aim to summarize the current animal models for SARS‐CoV‐2 infection, including mice, hamsters, nonhuman primates, and ferrets, and discuss the details of transmission, pathology, and immunology induced by SARS‐CoV‐2 in these animal models. We hope this could throw light to the increased usefulness in fundamental studies of COVID‐19 and the preclinical analysis of vaccines and therapeutic agents.

## Graphical abstract

Animal models that recapitulate characteristics and immune responses observed in COVID‐19 patients are urgently needed. These animal models such as mouse, hamster, nonhuman primate, and ferret, have provided robust platforms for studying the transmission, pathogenesis, and immunology induced by SARS‐CoV‐2, and for evaluating the immunomodulatory and antiviral drugs and vaccines against COVID‐19.

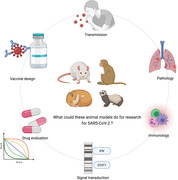

## 1 INTRODUCTION

Severe acute respiratory syndrome coronavirus 2 (SARS‐CoV‐2) with zoonotic origin emerges in 2019 and causes mild to severe respiratory illness in humans.^1^ The high transmission and mutation endow the ability of the novel coronavirus to develop unprecedentedly in the spatial range of epidemic areas. At the time of writing, the ongoing outbreak has caused over 200 million cases and more than 4 million deaths (https://covid19.who.int/). Clinical features are always initially characterized by fever, fatigue, dry cough, diarrhea, and chest pain.^1^, ^2^ The disease may progress to a severe SARS‐CoV‐2‐induced acute lung injury (ALI) and acute respiratory distress syndrome (ARDS), which causes the majority death in COVID‐19 patients.^3^ The lethal features include hyperinflammatory response, lymphopenia and microthrombosis, and so forth, leading to the diffuse alveolar damage and extensive pulmonary injury.^4^, ^5^ In addition, factors of age, cardiovascular disease, obesity, hypertension, and diabetes may advance the individual's risk when infected with SARS‐CoV‐2.^6^, ^7^, ^8^, ^9^

**FIGURE 1:**
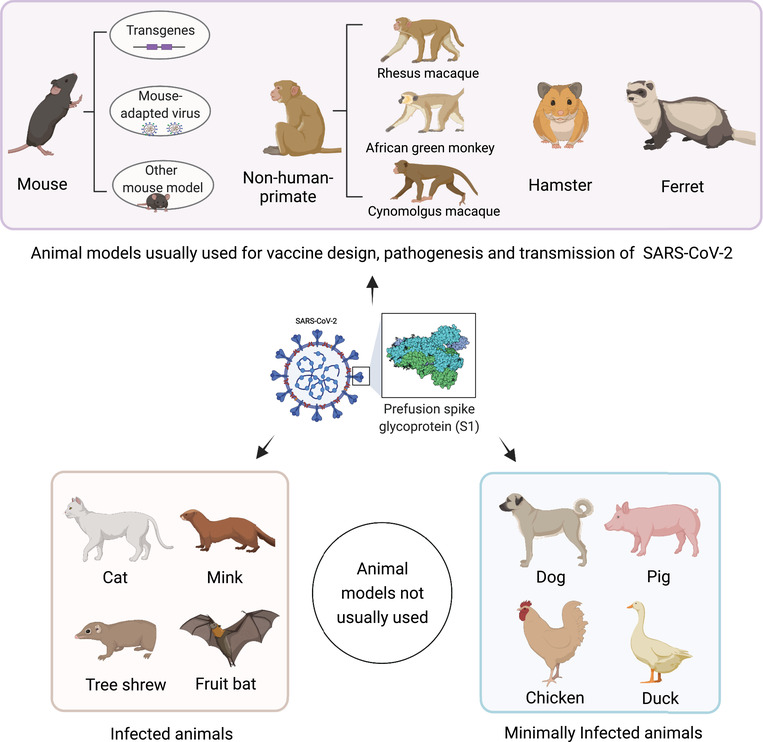
Animal models for SARS‐CoV‐2. Mouse, nonhuman primates (rhesus macaque, African green monkey, and cynomolgus macaque), hamster, and ferret are most used for studying the transmission, pathogenesis, and medical countermeasures. Mouse requires genetically modification for introducing hACE2 or adapted virus by serial passaging for SARS‐CoV‐2 infection. Animals of cat, mink, fruit bat, and Chinese tree shrew are susceptible to the virus infection, while dog, pig, chicken, and duck have the minimal infections; these animals are not usually used as models for SARS‐CoV‐2. The figure is created with BioRender.com

Testing medical countermeasures, including therapeutic agents and vaccines that reduce COVID‐19 morbidity and mortality, becomes an emergency scientific event.^10^, ^11^ Advancing studies on different animal models for COVID‐19 are imperative for this effort, and provide measurable readouts for the pathogenic mechanisms and potential interventions. To this end, several animal species, including mice, nonhuman primates (NHPs), hamsters, ferrets, and cats, have been well utilized in the studies for SARS‐CoV‐2 (Figure 1). In this review, we provide an overview of the current literatures on animal models for SARS‐CoV‐2 infection and summarize the features for harmonizing and improving the use of current models to investigate COVID‐19. We hope this will throw light to the preclinical analysis of vaccines and therapeutic agents.

## 2 MOUSE MODELS

**FIGURE 2:**
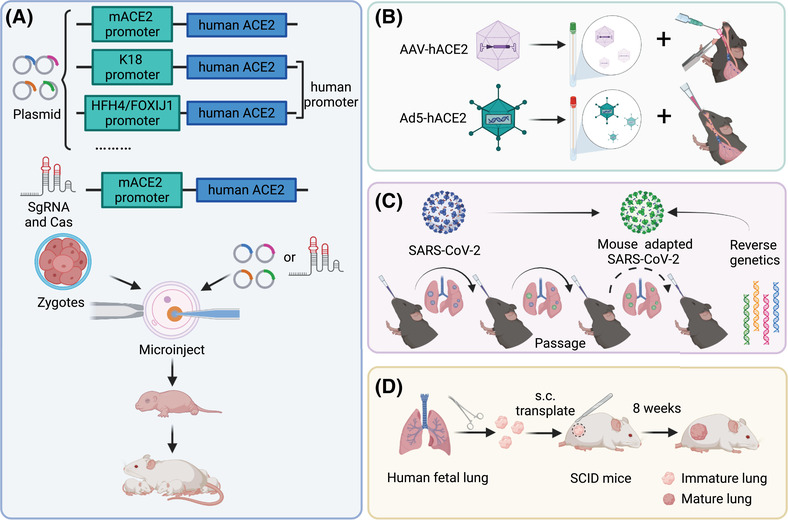
Strategies applied to the infection of mice. (A) Permanent genetic modifications are performed to introduce hACE2 to mice via CRISPR/Cas9 knock‐in technology or recombinant plasmid. The hACE2 expression is under the control of mouse ACE2 (mACE2) promoter, human epithelial cell cytokeratin‐18 (K18) promoter, or human lung ciliated epithelial cell‐specific HFH4/FOXJ1 promoter. (B) Ad5‐hACE2 or AAV‐hACE2 which expresses hACE2 is introduced to mice and sensitizes the respiratory tract of mice to SARS‐CoV‐2 replication. (C) Viruses are adapted to infect wide‐type mice by serial passaging or directly using reverse genetics. (D) SCID mouse‐human lung xenograft model of SARS‐CoV‐2 by surgically grafting human fetal lung tissue under the dorsal skin of SCID mice. The human lung xenografts can develop mature structures closely resembling the normal human lung and show a high viral replication with spreading to the whole lung tissue when infected with SARS‐CoV‐2. The figure is created with BioRender.com

Mouse (*Mus musculus*) model has been well used for many viral investigations, such as Middle East respiratory syndrome coronavirus (MERS‐CoV)^12^, ^13^ and SARS‐CoV.^14^, ^15^ The main impediment for SARS‐CoV‐2 infection in mice is the lack of proper receptors, because SARS‐CoV‐2 entering host cells mainly relies on the human angiotensin‐converting enzyme 2 (ACE2) receptor not mouse ACE2.^16^ Therefore, several strategies have been applied to resolve the problem, such as development of genetical modification and mouse adapted virus (Figure 2). We will detail the current mouse models used in SARS‐CoV‐2 investigation and discuss the pathogenic features below.

### 2.1 Genetically modified mice

Generation of human ACE2 (hACE2) receptor expressing mouse is considered as a forthright strategy for SARS‐CoV‐2 infection in mice. To date, several methods including introducing hACE2 into mice via adenoviral/adenoviral‐associated vectors, expressing hACE2 by mouse ACE2 promoter or heterologous gene promoters and clustered regularly interspaced short palindromic repeats (CRISPR) systems, have been used to develop genetically modified mice. Notably, the varied expression of hACE2 among different organs in mice has led to the mild to severe disease in these mouse models, adding further complexity as factor of investigation of SARS‐CoV‐2‐induced pathogenesis and evaluation of therapeutic agents and vaccines.

The first SARS‐CoV‐2 infected transgenic mice are Institute of Cancer Research (ICR) background and express hACE2 under the control of mouse ACE2 promoter.^17^ The mice intranasally infected with SARS‐CoV‐2 result in the weight loss, high viral replication in lung tissues, and exhibit interstitial pneumonia with infiltration of macrophages and lymphocytes.^17^ The humoral immunity is induced on 21 days postinfection (dpi). Neurological symptoms such as anosmia, confusion and encephalopathy,^18^, ^19^ and cardiac injury and myocarditis^20^, ^21^, ^22^ are common features that have been reported in COVID‐19 patients. However, the virus is not observed in other organs in this mouse model, such as the heart and brain, except for the intestine.^17^ Using CRISPR/Cas9 knock‐in technology, hACE2 linking to tdTomato gene is inserted to the first coding exon of mouse ACE2 by homologous recombination, and the expression is driven by the mouse ACE2 promoter.^23^ The hACE2 mice are susceptive to the intranasal infection of SARS‐CoV‐2 and present robust viral replications in organs of lung, trachea, and brain, as well as the olfactory epithelium, and develop the interstitial pneumonia and olfactory dysfunction accompanied by elevated cytokines.^23^, ^24^ Notably, the aged mice show marked decrease in weight loss and more severe pathology with increased neutrophil infiltration than those in the young ones, consistent with the clinical symptoms observed in COVID‐19 patients.^23^ In one study, researchers intratracheally instill SARS‐CoV‐2 to the mice with CRISPR/Cas9 knock‐in of hACE2.^25^ The hACE2 mice develop severe pulmonary pathologies with hemorrhage, inflammatory cell infiltration and hyaline membrane formation, and so forth, recapitulating many features observed in COVID‐19 patients with ARDS.^25^ Recently, a strategy of rapid generation of hACE2 mice has been developed via combining CRISPR‐Cas9 system with tetraploid complementation.^26^ The hACE2 gene is inserted into exon 2 and the expression is driven by the mouse ACE2 promoter. The mice show high viral replication in lung, airway, and small intestine. Pulmonary injury, such as alveolar septal thickening, inflammatory cell infiltration, hemorrhage, and hyaline membrane formation, are observed in mice with background of C57BL/6 or BALB/c, but the severest injury occurs earlier in the BALB/c mice,^26^ indicating an earlier inflammatory response.

K18‐hACE2 transgenic mice, in which hACE2 expression is driven by the human epithelial cell cytokeratin‐18 (K18) promoter,^27^ has been used for studying the pathogenesis of SARS‐CoV^28^ and SARS‐CoV‐2^29^ and can cause lethal infection. K18‐hACE2 mice exhibits high susceptible to SARS‐CoV‐2 infection. Intranasal inoculation of SARS‐CoV‐2 results in an approximate 25% of weight loss of mice along with virus spreading to other organs of heart, brain, kidney, spleen, duodenum, and colon.^30^, ^31^, ^32^, ^33^ High levels of viral infection in lung tissues severely impair the pulmonary structure and function, and cause thrombosis and vasculitis.^30^, ^31^, ^34^ Severe infiltration of activated neutrophils, monocytes and lymphocytes to lungs, and dysregulated type I/ II IFN response are observed during SARS‐CoV‐2 infection in K18‐hACE2 mice.^30^, ^35^ Cytokine storm directly correlates with pulmonary injury, multiple organ failure, and an unfavorable prognosis, resulting in ARDS and systemic inflammatory response syndrome (SIRS).^36^ Cytokine storm has been found in the lung, spleen, and even in the brain of K18‐hACE2 mice after intranasal inoculation with SARS‐CoV‐2, leading to multiple organ injury.^32^, ^37^ K18‐hACE2 mice could develop anosmia at early time points after infection,^34^ followed by virus neuroinvasion that occurs in mice through the olfactory neuroepithelium or eye in a manner that virus transports only partially depending on hACE2,^32^, ^38^ a mechanism observed in COVID‐19 patients.^39^ Interestingly, the male mice show a higher lethality compared to the female ones, perhaps because of the association with the higher level of inflammatory cytokines.^31^ Notably, isolations of SARS‐CoV‐2 in lung and brain tissues from infected K18‐hACE2 mice show differences in genetics and plaque morphology; inoculation of these of lung and brain SARS‐CoV‐2 isolations into new batches of K18‐hACE2 mice results in the lethal pulmonary and central nervous system infection, respectively.^33^ The highly tendentious pathogenicity of genetic variants of SARS‐CoV‐2 in different organs may provide an alternative approach to investigate the specific features observed in COVID‐19 patients. Recently, lethal diseases have also been reported among K18‐hACE2 mice infected with SARS‐CoV‐2 variants (B.1.1.7 and B.1.351),^40^ suggesting that K18‐hACE2 mice can be used as a robust mouse model against the rapid evolution of SARS‐CoV‐2. Therefore, K18‐hACE2 transgenic mice have been widely used in the preclinical evaluation of vaccines^41^, ^42^, ^43^, ^44^ and antiviral drugs^45^, ^46^, ^47^, ^48^ against severe SARS‐CoV‐2 infection and COVID‐19 disease.

Another genetically modified mice, that is, HFH4‐hACE2 mice, express hACE2 under the control of a lung ciliated epithelial cell‐specific HFH4/FOXJ1 promoter.^49^, ^50^ HFH4‐hACE2 mice have been used for investigating the SARS‐CoV^50^, ^51^ and SARS‐CoV‐2 pathogenesis.^52^, ^53^ The hACE2 expression level is varied among different organs and is higher in lung tissues.^54^ Intranasal infection of SARS‐CoV‐2 induces high viral replication in lung tissues of HFH4‐hACE2 mice, and causes interstitial pneumonia accompanied by elevated cytokines and infiltration of macrophages and lymphocytes and fibrin exudation.^54^ The HFH4‐hACE2 mice are also susceptive to the infection of SARS‐CoV‐2 with D614G mutation.^55^ Notably, two infection outcomes have been observed: (1) those that lost more than 20% of body weight often accompanied by noticeable neurological symptoms and died eventually; (2) those that lost less than 10% of body weight and survived. Moreover, the HFH4‐hACE2 mice exhibit a gender‐dependent progression that the males develop more lethal disease,^54^ sharing some features observed in K18‐hACE2 mice. Besides, the preexposure to SARS‐CoV‐2 protects HFH4‐hACE2 mice from severe pneumonia,^54^ indicating an adaptive immunity response in mice. Nevertheless, only some limited studies evaluate the therapeutic agents and vaccines using the HFH4‐hACE2 transgenic mice.^53^, ^56^

The CAG‐hACE2 transgenic mice have ever been used for investigating the SARS‐CoV infection.^57^, ^58^ A recent study reports the generation of CAG‐hACE2 mice, which express HA‐tagged hACE2 drove by the CAG promoter and is flanked by HS4 insulators.^59^ High level of hACE2 expression in organs of lung, brain, and kidney endows the CAG‐hACE2 mice the susceptibility to the SARS‐CoV‐2 infection.^59^ Intranasal infection with low dose virus (1000 plaque‐forming units, PFU) results in an absolute mortality with severe pulmonary pathologies, and the male CAG‐hACE2 mice exposed to SARS‐CoV‐2 infection exhibit much more weight loss than the female mice,^59^ which is consistent with the sex‐biased response observed in K18‐hACE2 mice and HFH4‐hACE2 mice as described above. In addition, another study reports the C57BL/6‐Tgtn Smoc mice, which express hACE2 that links to luciferase driven by the CAG promoter (CAG‐human ACE2‐IRES‐Luciferase‐WPRE‐polyA), as models for SARS‐CoV‐2 investigation. The hACE2 expression is high in organs of heart, kidney, stomach, lung, spleen, and liver. Intranasal inoculation with 3 × 10^4^ PFU of SARS‐CoV‐2 induces viral replication in lung and intestine tissues, and causes the interstitial pneumonia characterized by alveolar septa thickening and interstitial infiltrates.^60^ Some medical countermeasures such as vaccines and antiviral drugs are under evaluation using the CAG‐hACE2 mice.^60^, ^61^

Finally, instead of permanent genetic modification, adenovirus or adeno‐associated virus that expresses hACE2, that is, Ad5‐hACE2 or adeno‐associated virus (AAV)‐hACE2 which can transduce a large proportion of pulmonary epithelial cells, is introduced to mice to sensitize the respiratory tract for SARS‐CoV‐2 replication. The wide‐type mice intranasally^62^ or oropharyngeally^63^ inoculated with Ad5‐hACE2 show high hACE2 expression confining to the alveolar epithelium and occasional expression in the airway epithelium. The transient effect of hACE2 expression can sustain for several days for SARS‐CoV‐2 infection and induces high viral replication in lung tissues, which terminally results in a rapid 20% of weight loss and impairs the pulmonary functions.^62^, ^63^ A variety of lesions including inflammatory infiltrates, hemorrhage, alveolar edema, and necrotic cell debris with markedly elevated cytokines such as TNF‐α and IL‐6, are observed.^62^, ^63^, ^64^ Type I IFN response is observed during SARS‐CoV‐2 infection in Ad5‐hACE2‐transduced mice, accompanied by upregulation of signal transducer and activator of transcription 1 (STAT1) signaling, which is critical in protection against SARS‐CoV‐2.^62^, ^63^ Interestingly, pulmonary endothelial cells show viral replication and are induced endotheliopathy in Ad5‐hACE2 mice^65^; other organs of liver and intestine, and so forth, do not exhibit viral replication.^62^, ^63^ The responses between C57BL/6 mice and BALB/c mice, such as the weight loss, viral replication, and the degree of pulmonary lesson, are slightly different.^62^ In a recent study, intranasal inoculation of Ad5‐hACE2 causes the SARS‐CoV‐2 infection in peripheric leukocytes of mice as early as 1 dpi, which contributes to the systemic viral dissemination.^66^ AAV‐mediated expression of hACE2 in mice also endows the viral replication in lung tissues, and increases the pulmonary infiltration of monocytes, neutrophils and lymphocytes, and Type I IFN response and humoral immunity.^67^, ^68^ However, adenovirus‐mediated delivery results in viral replication to lower titers and mild clinical signs of infection compared to those of K18‐hACE2 mice.^69^ The adenovirus or adeno‐associated system circumvents the problem of time‐consuming process of breeding multiple generations and represents an easily and rapidly strategy for the currently urgent need of small animal models for COVID‐19. Countermeasures of new therapies and vaccines against SARS‐CoV‐2 have been under evaluation using the Ad5‐hACE2‐ or AAV‐hACE2‐based mouse model.^62^, ^70^, ^71^, ^72^, ^73^ A limitation of these mice is that hACE2 is expressed ectopically, which may change the tissue or cellular tropism of the virus.

### 2.2 Mouse‐adapted viruses

Virus can be adapted to infect wide‐type mice by serial passaging, which may more closely resemble the natural host‐pathogen interactions in mice. This strategy is successful because of a swarm of related viral quasi species in the populations of RNA viruses. The rare viruses in the swarm that contain mutations in the key protein that increase their binding affinity to entering receptors of mice are expected to be selected, owing to their higher levels of replication in lung tissues. This strategy has been successfully applied to investigate the SARS‐CoV^14^, ^74^ and MERS‐CoV,^12^ and has generated highly infective viruses and caused lethal pulmonary diseases.

Recently, evolution in vivo via serial passage of SARS‐CoV‐2 in the mouse lung tissue generates highly infective virus, that is, MASCp6, with increased virulence in both young and aged mice.^75^ The mice intranasally inoculated with MASCp6 develop the interstitial pneumonia and inflammatory response. MASCp6 infection causes severe pulmonary pathology and higher inflammation in the aged mice than those in young ones.^75^ Also, MASCp36, derived from MASCp6 virus by serial passaging again, obtains robust affinity to mouse ACE2 and exhibits age and gender‐related skewed distribution of mortality with 50% of mortality in aged, male mice when inoculation with only 100 PFU of MASCp36.^76^ Another mouse‐adapted virus, that is, SARS‐CoV‐2 MA10, recapitulates the age‐related disease severity observed in humans as well, and causes lethal features of ALI and ARDS, and death in wild‐type mice.^77^ Interestingly, BALB/c mice suffer more sorrows compared with C57BL/6J mice, which may because of the different immune paradigms induced by the virus in these two background mice. Other mouse‐adapted SARS‐CoV‐2 viruses, such as HRB26M,^78^ MACo3,^79^ and rSARS2‐N501Y_P30_,^80^ have been reported the caused moderate to lethal disease.

In addition, some researchers have directly developed mouse‐adapted virus using reverse genetics, including SARS‐CoV‐2 MA (precursor of SARS‐CoV‐2 MA10)^81^ and rSARS2‐N501Y_P0_ (precursor of rSARS2‐N501Y_P30_).^80^ These mouse‐adapted viruses can replicate in the upper and lower airways of mice; however, the viruses are only able to cause mild disease in mice even intranasal inoculation with a high dose of the adapted viruses. Therefore, evolution in vivo via serial passaging of modified SARS‐CoV‐2 using reverse genetics may be an ideal approach to develop SARS‐CoV‐2 strain with high infective and lethal to the general laboratory mice.

Some trials including potential medical countermeasures against SARS‐CoV‐2, have been in progress or done using the mouse model with mouse‐adapted virus.^82^, ^83^, ^84^ However, the mouse model has limitations: (1) The mouse‐adapted virus may cause the infection that does not recapitulate many aspects of human disease and may induce injury via unique pathogenesis; (2) The occurrence of key mutations in immunogen such as receptor‐binding domain (RBD) which is the primary target for the neutralizing antibody response, may lead to the noneffective neutralizations for the mouse‐adapted virus.

### 2.3 Other mouse models

Several studies have applied the immunodeficient mouse to investigate the pathogenesis in human‐specific viral infections, such as SARS‐CoV.^85^, ^86^ Recently, researchers have developed a severe combined immunodeficiency (SCID) mouse‐human lung xenograft model for SARS‐CoV‐2 by surgically grafting human fetal lung tissue under the dorsal skin of SCID mice.^87^ The human lung xenografts can develop mature structures closely resembling the normal human lung and show a high viral replication with spreading to the whole lung tissue when injected with SARS‐CoV‐2.^87^ The SARS‐COV‐2 infection in this mouse model confines to epithelial cells and causes a severe pulmonary damage with elevated pro‐inflammatory response.^87^ The immunodeficient mouse can also be used for studying the role of immune effectors combining the human immune system and ACE2 expression; however, this strives has not been used for evaluating the vaccines and therapies.

**FIGURE 3:**
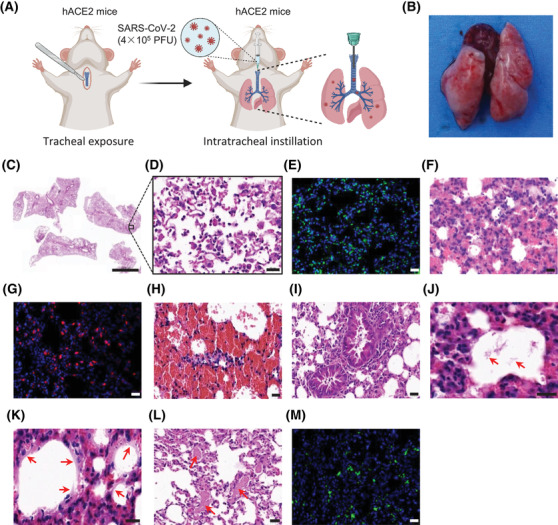
The pulmonary features of a mouse model for SARS‐CoV‐2‐induced ARDS. (A) An illustration for route of the establishment of a mouse model for ARDS. (B) The gross observation for pulmonary morphology on 5 dpi. (C–D) Images of H&E staining for pulmonary pathological changes of mice on 5 dpi. (E) Images of TUNEL staining for cell apoptosis in lung tissue on 5 dpi. (F–G) Images of H&E staining (F) and immunofluorescence staining for Ly6G (G) indicate the neutrophils as the predominant cell infiltration with 60% of the lung tissue. (H–L) SARS‐CoV‐2 infection induces apparent hemorrhage (H), inflammatory cells (I), proteinaceous debris (J, red arrow), hyaline membranes‐like changes (K, red arrow), and thrombi (L, red arrow) in the lung tissue of the mouse model. (M) Images of immunofluorescence staining for SARS‐CoV‐2 spike protein in the lung section of a mouse with SARS‐CoV‐2‐induced ARDS. Scale bars represent 2000 μm (C) or 20 μm (D‐M). Reprinted with the permission from a published reference^25^

The high mortality of SARS‐CoV‐2 infection closely associates with the disease progression to a severe COVID‐19‐induced ARDS.^88^ The mortality rate in COVID‐19 patients with ARDS is up to 50% and reaches 94% in those who received mechanical ventilation.^9^ To this end, mouse models that recapitulate the features of COVID‐19 patients with ARDS should be highlighted. We have ever established a mouse model via intratracheal instillation of SARS‐CoV‐2 (4 × 10^5^ PFU) to hACE2 mice (Figure 3).^25^ The transgenic mice are generated using CRISPR/Cas9 knock‐in technology. Severe gross lesions involve the bigger size with the bilateral congestion and edema, and patched hemorrhage of lung on 5 dpi. The infected mice develop severe bronchopneumonia, diffuse interstitial pneumonia, and vasculitis. Histopathological changes in lung tissue include reduction of airway space, thickened alveolar septa, pulmonary edema, necrosis, proteinaceous debris in the alveolar space, formation of hyaline membrane, occasional thrombus in pulmonary capillary lumen, consolidation, diffuse hemorrhage, and alveolar damage. Inflammatory infiltrates include massive neutrophils, and scattered monocytes, macrophages, and lymphocytes throughout the lung tissue. Notably, the neutrophils are infiltrated to lung as early as 6 h post inoculation of SARS‐CoV‐2, and present the predominant cell infiltration until 3 dpi. The dead cells are mostly shown in the background of the inflammatory cell infiltrates. In addition, a recent study has reported an animal model for SARS‐CoV‐2‐induced ARDS in genetically unaltered CD‐1 mice.^89^ Bleomycin or ricin‐pretreated mice show highly susceptible to SARS‐CoV‐2 infection, accompanied by sustained body weight loss and more than 50% of mortality rates. Notably, the viral replication is observed in the upper and lower respiratory tracts and in the organs of lung and heart, and in serum samples. The deleterious effects induced by SARS‐CoV‐2 infection include severe pulmonary damage characterized by extensive peribronchial and perivascular inflammatory cell infiltrates. This genetically unaltered mouse might be infected via a novel mechanism other than the canonical ACE2‐mediated uptake route. Besides, some strategies, such as intratracheal instillation of Poly I:C and SARS‐CoV‐2 spike protein^90^ and intravenous injection of SARS‐CoV‐2 envelope (2‐E) protein,^91^ have also been reported to induce ARDS‐like pathological damages in genetically unaltered mice. Finally, the K18‐hACE2 mice can develop lethal disease after SARS‐CoV‐2 infection as describe previously, and may be an alternative model for the ARDS‐like pathological investigation.

Chronic diseases, such as obesity, cardiovascular disease, hypertension, and diabetes, are closely correlated to the severe COVID‐19 disease and advance the SARS‐COV‐2‐induced mortality.^7^, ^8^, ^9^ Genetic or dietary modification that induces features of these human chronic diseases has been implemented in mice.^92^, ^93^, ^94^ Efforts of developing the risk‐related models for studying SARS‐CoV‐2 infection may involve mouse‐adapted SARS‐CoV‐2 virus or mice humanized with hACE2 combining related chronic diseases. For example, low dose of mouse‐adapted SARS‐CoV‐2 which is generated through serial passing in the mouse lung, has been reported to cause the enhanced morbidity in aged and diabetic obese mice (generated by dietary modification), resembling the clinical features observed in COVID‐19 patients.^95^ Nevertheless, these chronic disease‐related mouse models for SARS‐CoV‐2 infection need to be fully explored, which may provide a robust platform for the efficiency evaluation of potential vaccines and drugs to combat with SARS‐CoV‐2 infection.

Although appropriate mouse models for studying SARS‐CoV‐2 transmission have not been reported, the emerged variants might impart cross‐species transmission of SARS‐CoV‐2 to naïve mice by enhancing receptor binding, such as the currently reported N501Y, E484K, and K417N variants.^96^, ^97^, ^98^ The mutations render effective binding of the RBD to mouse ACE2 and lead to fusion of neighboring membranes and effective infection,^97^ which have been found in the mouse‐adapted viruses as well, especially the N501Y mutation.^75^, ^95^ Further investigations on the potential implications for SARS‐CoV‐2 transmission, such as mouse‐to‐mouse, or mouse as an intermediate host, need to be made to clarify the possible routes whereby viruses are transmitted and limit the mouse‐mediated transmission.

In summary, several mouse models have been used or under development for investigating the pathogenesis of SARS‐CoV‐2 infections. These mouse models develop mild to lethal diseases; however, none of these models can replicate all features observed in COVID‐19 patients, especially the unusual aspects such as the pulmonary vascular disease. Furthermore, more efforts on using mouse models need to be made to demonstrate the roles of risk‐related factors, such as cardiovascular disease and obesity in SARS‐CoV‐2 infection.

## 3 SYRIAN HAMSTER MODELS

Syrian golden hamsters (*Mesocricetus auratus*) are small mammals that have been used for decades to study the infections of human respiratory viruses, including influenza virus, SARS‐CoV,^99^, ^100^, ^101^ and genetically modified animals generated for MERS‐CoV.^102^ The ACE2 domain of human that interacts with SARS‐CoV‐2 RBD of S protein closely resembles that of hamsters,^103^ suggesting that hamsters could be suitable as a natural model for studying the SARS‐CoV‐2 transmission and infection. As expectedly, intranasal inoculation with 5 × 10^4^ TCID50 (median tissue culture infective dose) of SARS‐CoV‐2 induces approximately 15% of weight loss and viral replication in upper and lower respiratory tracts and organs of lung, heart, brain, spleen, and liver of hamsters.^104^, ^105^ The virus in the lung tissue is mainly presented in the bronchial epithelium, macrophages, and alveolar epithelial cells (type I and II), followed by rapid viral clearance between 6 and 7 dpi.^105^, ^106^ The hamsters develop mild clinical scores and recover on 14 dpi.^105^, ^106^ However, intranasal inoculation with high dose of SARS‐CoV‐2 (5 × 10^5^ TCID50) induces a severe pulmonary injury in hamsters that shares many features with COVID‐19 patients, including bilateral and multilobular ground glass opacity, severe weight loss, and increased mortality.^104^, ^107^, ^108^ Multiple organ damages are also observed in the severe SARS‐CoV‐2 infected hamster model, accompanied by higher level of viral replication in tissues of lung, heart, brain spleen, liver, lymph node, kidney, adrenal gland, different segments of alimentary tract, ovary, prostate, and vesicular gland, which eventually leads to the systemic injury.^108^ Notably, the focal lesions still exist in part tissues of liver, lymph node, and heart after prolonged infection,^108^ suggesting a sustaining damage. The diseases of genital system and digestive system induced by SARS‐CoV‐2 in hamsters would provide a suitable aspect in related human diseases. Besides, olfactory epithelium is also damaged by SARS‐CoV‐2 infection, resulting in the olfactory dysfunction in hamsters as early as 2 dpi.^109^, ^110^, ^111^ In one study, hamsters are orally inoculated with SARS‐CoV‐2 (1 × 10^5^ PFU) and develop no sign of disease compared to the equal viral quantity of intranasal inoculation.^112^ The viral replication in organs of lung and small intestine are much lower and the body weight shows no change,^112^ suggesting the respiratory infection with less efficiency when oral acquisition of SARS‐CoV‐2 in hamsters.

In hamsters, SARS‐CoV‐2 infection causes mild to lethal pathologic changes. Multifocal bronchiolar epithelial necrosis and inflammatory infiltrates of lung occur as early as 2 dpi,^104^ consisting of many neutrophils, macrophages, monocytes, and a few lymphocytes, and develop the most severe on 4 dpi.^105^, ^106^, ^113^ The infection can progress to interstitial pneumonia characterized by massive pulmonary consolidation after infection.^104^, ^105^, ^106^ In addition, endothelialitis, alveolar hemorrhage, and pulmonary edema are also observed in severe infected hamsters.^105^, ^114^ Infiltration of lymphocytes, pneumocyte hyperplasi, and fibrosis are main features in the later progression.^105^, ^114^

SARS‐CoV‐2 infection in the hamster model is a closely gender‐ and age‐dependent progression. The old hamsters show more pronounced weight loss and severe pulmonary disease, such as alveolar and perivascular edema, more pulmonary inflammatory infiltrates, and consolidation, compared to the young ones when infected with SARS‐CoV‐2.^106^ However, the response to the infection is quite different, that the young hamsters exhibit earlier viral replication and spreading and immune cell influx to lung, while the old hamsters show a delayed and more adverse response to the SARS‐CoV‐2 infection.^106^ Resembling the age‐dependent progression, male hamsters show a high viral replication, weight loss, and severe pulmonary disease.^114^ The manifestations are coincident with those of COVID‐19 patients.

Importantly, the hamsters are suitable as models to investigate the transmission of SARS‐CoV‐2. SARS‐CoV‐2 can be transmitted by direct contact or via aerosols (respiratory droplets or airborne droplet nuclei) between humans. To date, several studies have launched this animal model for the efforts to prevent the SARS‐CoV‐2 transmission. For example, SARS‐CoV‐2 can be transmitted efficiently from infected hamsters to naive hamsters via aerosols or by direct contact; using surgical mask could well reduce the infected proportion of hamsters (from 66.7% to 16.7%),^115^ suggesting the potential benefit of using surgical mask against the SARS‐CoV‐2 transmission. Intranasal inoculation of SARS‐CoV‐2 causes the viral replication in epithelial cells of the duodenum and viruses in faeces, suggesting the hamsters as models for studying the oral‐faecal route of transmission of SARS‐CoV‐2.^105^ Notably, transmission via fomites such as soiled cages is not as efficient and the communicable period is short and closely correlates with the infectious virus.^105^ However, unlike the challenged hamsters, infected naive hamsters show minimal clinical symptoms, lower viral replication in respiratory tract, and milder disease, and can recover to the original condition. Therefore, the infected naive hamsters may not fully recapitulate the features observed in SARS‐CoV‐2‐infected humans.

Expression levels of chemokines and cytokines, which link to COVID‐19 in humans including IL‐6, IL‐10, IFN‐λ, IFN‐γ, CXCL‐10, MX‐2, and TNF‐α, and so forth, are robustly increased in hamsters on 4 dpi, and are gradually resolved on 7 dpi.^116^, ^117^, ^118^ Type I and III IFN signaling has been involved in the restricting infection of many respiratory viruses including SARS‐CoV and SARS‐CoV‐2.^118^, ^119^ In STAT2‐knockout hamsters, the immune responses including Type I and III IFN signaling are decreased with a reduced injury of immune pathology; however, the viral replication is increased and disseminate to several peripheral tissues without the keeper of STAT2.^118^ Besides, a study on RAG2‐knockout hamsters shows that the absence of the functional B and/or T cells can exacerbate pathology at the early stage of SARS‐CoV‐2 infection.^120^ IL‐2 receptor knockout in hamsters results in the prolonged viral persistence, suggesting the susceptibility of SARS‐CoV‐2 to adaptive immune control.^113^ Like the observations in COVID‐19 patients, adaptive response is induced and neutralizing antibodies are produced as early as 7 dpi in hamsters.^105^, ^121^ Passive immunization or antibody therapy shows benefits of reduction of viral dissemination and progression in hamsters.^107^, ^120^, ^122^, ^123^ Therefore, the hamster model may be used as an alternative platform to investigate the immune response, therapies, and vaccines.

## 4 NHP MODELS

**FIGURE 4:**
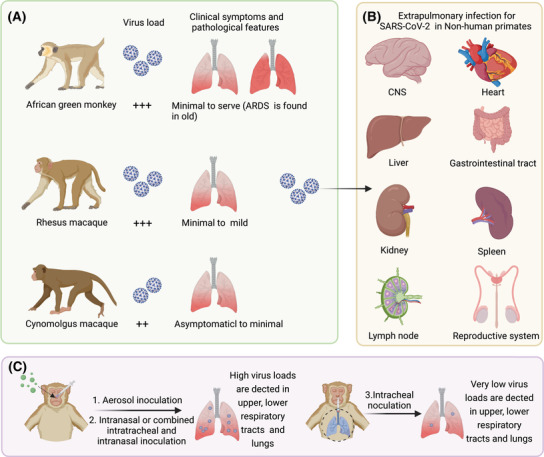
SARS‐CoV‐2 infection in nonhuman primates. (A) Rhesus macaques, African green monkeys, and cynomolgus macaques are all susceptive to SARS‐CoV‐2 infection. Asymptomatic to minimal clinical features and minimal to mild clinical features are observed in cynomolgus macaques and rhesus macaques, respectively. The African green monkeys have a spectrum of responses from minimal to severe disease, in which the aged could progress to ARDS. (B) Viral replication is observed in extrapulmonary organs including brain, spleen, liver, heart, kidney, gastrointestinal tract, uterus, urogenital tract, and lymph node. (C) The route of aerosol, intranasal, or combined intratracheal and intranasal inoculation of SARS‐CoV‐2 causes high viral loads in the upper and lower respiratory tracts and lungs in nonhuman primates. Only intratracheal inoculation causes much lower viral loads in the upper and lower respiratory tracts and lungs. The figure is created with BioRender.com

NHPs are desirable models with similar anatomy, phylogeny, and immunology to humans. There are a number of researches that have applied those NHP modes, including rhesus macaques (*Macaca mulatta*), African green monkeys (*Chlorocebus aethiops*), and cynomolgus macaques (*Macaca fascicularis*), for investigating the pathogenesis involved in SARS‐CoV‐2 infection and also for developing the medical countermeasures (Figure 4). The ACE2 protein in NHP especially in rhesus macaques gives an overall homology of 91% compared with hACE2.^124^ Analysis of NHP single‐cell RNA‐sequencing data sets reveals that the cell subtypes of type II pneumocytes, nasal goblet secretory cells, and absorptive enterocytes co‐express ACE2/type II transmembrane serine protease 2 (TMPRSS2),^125^ suggesting the potential as cellular targets of SARS‐CoV‐2. Previous studies have demonstrated that African green monkeys are more susceptible to the SARS‐CoV infection than rhesus and cynomolgus macaques that cause severe disease.^126^ The species differences are slightly varied when infected with SARS‐CoV‐2. For example, SARS‐CoV‐2 infection have been reported to cause only minimal to mild clinical features, such as reduced appetite, dehydration, transient fever (occurs between 1 and 2 dpi), mild weight loss, and occasional coughing, with peaking on 7 days in rhesus macaques and recovery between 9 and 17 days responding to SARS‐CoV‐2 infection^127^, ^128^; the African green monkeys have a spectrum of responses from minimal to severe disease,^129^, ^130^, ^131^ while the cynomolgus macaques have a milder decreased physical activity or asymptom.^132^, ^133^, ^134^ Rhesus macaques, African green monkeys, and cynomolgus macaques are all susceptible to the SARS‐CoV‐2 infection, but differences in time‐shifted viral replication and clearance are found among the three kinds of NHPs.^132^, ^133^, ^134^ Intranasal or combined intratracheal and intranasal inoculation of SARS‐CoV‐2 induces the robust viral replication in the upper respiratory tract including nasal turbinate and throat, with peaking between 1 and 3 dpi and persisting until 5–7 days before dropping below detection limits between 14 and 18 dpi.^135^ In two studies, SARS‐CoV‐2 infection causes a second wave of viral replication in nasal swab samples on 5 dpi, with prolonged viral shedding period in nasal tissues that can persist for 4 weeks.^128^, ^136^ The viral replication in the lower respiratory tract such as trachea and in lung tissues increases on 3 dpi and peaks on 9 dpi in one study.^128^ Extrapulmonary organs including brain, spleen, liver, heart, kidney, eye, gastrointestinal tract, uterus, bladder, central nervous system, urogenital tract, and lymph node, are also observed viral replication, suggesting the potential of systemic injury.^128^, ^134^, ^135^, ^137^, ^138^, ^139^, ^140^ However, some studies negate the viral replication in extrapulmonary organs, except for gastrointestinal tract and lymph node.^127^ Notably, only intratracheal inoculation of SARS‐CoV‐2 does not induce the viral replication in nasal tissue and much lower viral loads are observed in the nose and throat swab and in lung tissue compared to the route of intranasal or combined intratracheal and intranasal inoculation.^141^ The difference suggests that nasal infection congregates more virus in the upper respiratory tract and aggravates the viral transmission and infection in the lower respiratory tract that may cause more severe disease. Besides, investigators also use the LMA mucosal atomization device that resembles the human infection; this route causes the similar viral replication expect for the prolonged viral loads compared to the nasal infection.^142^ In aged NHPs, the viral replication in the upper respiratory tract persists for 10 days with peaking loads and is much higher in lung tissues compared to the young ones^143^; while the gender might be not the risk factor in severe disease.^134^

In NHPs, SARS‐CoV‐2 infection causes minimal to severe pathologic changes. Inflammation in the upper airways is characterized by multifocal squamous metaplasia of the respiratory epithelium and infiltration of a small number of neutrophils. Severe gross lesions involve pulmonary punctate hemorrhage and red lesion.^128^, ^134^, ^142^ Bronchopneumonia and interstitial pneumonia occur as early as 3 dpi that frequently center on small bronchus and terminal bronchioles, respectively.^127^, ^136^ Multifocal clusters of virus‐infected cells are presented throughout the pulmonary parenchyma.^144^ Histopathological changes in lung tissues include thickened alveolar septa, diffuse alveolar damage, formation of hyaline membrane, occasional diffuse hemorrhage, collagen deposition, pulmonary edema, consolidation, minimal necrosis, and pneumocyte hyperplasia. One study has shown the thrombus in pulmonary capillary lumen accompanied by endothelial cell swelling and perivasculitis.^136^ Inflammatory infiltration of monocytes, macrophages, neutrophils and eosinophils aggregates in alveoli, pulmonary parenchyma and in the respiratory epithelial submucosa of larger airways, and a small number of lymphocytes are confined predominantly to perivascular sites.^142^ SARS‐CoV‐2 antigen is presented in NHPs associated with pulmonary lesions, especially in the alveolar epithelial cells and macrophages.^142^, ^143^ The NHPs develop the most severe pathology between 4 and 5 dpi, and the diseases of those alleviate gradually but display moderate to mild alveolar inflammation.^128^, ^133^ The infection causes long‐standing pulmonary injury accompanied by multifocal chronic interstitial pneumonia even on 34 dpi.^142^ However, some in vivo studies demonstrate the recovery undergo SARS‐CoV‐2 infection in NHPs.^127^, ^137^ Extrapulmonary tissues, such as brain, heart, liver, spleen, kidney, stomach, ileum, colon, testis, and lymph node, have been reported to present mild histopathological lesions.^128^, ^134^, ^136^ Those organs show varied levels of edema, infiltration of inflammatory cells on the early stage of infection, while the sustained systemic inflammatory response is devoid. For example, microhemorrhages and neuropathology that is consistent with hypoxic injury are presented in SARS‐CoV‐2‐infected NHPs.^145^ Besides, lymph nodes of mesenteries and lung, and spleen display increased immune activity by enlarging germinal centers in response to viral infection.^128^, ^134^ Like the observations in COVID‐19 patients, the aged NHPs develop more severe diffuse interstitial pneumonia compared to the young ones,^130^, ^143^ and the aged African green monkeys are reported to progress to ARDS,^130^ which has been noted as the main cause of mortality induced by SARS‐CoV‐2. These findings highlight the importance of including age in the selection of animals.

Involved chemokines and cytokines including IL‐1Ra, IL‐1β, IFN‐γ, IFN‐α TNF‐α, IL‐6, IL‐2, IL‐4, IL‐5, RANTES, G‐CSF, GM‐CSF, CCL2, and IP‐10, and so forth, are increased at a range scale of time and space.^128^, ^130^, ^133^, ^134^, ^135^ Activation of innate immune response occurs between 1 and 3 dpi accompanied by type I IFN response; then the Th1/Th2 response and adaptive response are induced at the middle and late stage of infection. Neutralizing antibodies are produced as early as 5 days after SARS‐CoV‐2 infection in NHPs and peak between 15 and 21 dpi.^127^, ^135^ The induced immune response protects those of NHPs from adverse effects when rechallenged the SARS‐CoV‐2, including lower viral replication, no remarkable lesion in the upper and lower respiratory tracts and no significant bronchopneumonia, and produces significantly higher neutralizing antibody titers.^136^, ^137^, ^144^ Notably, the aged NHPs present a delayed immune response with more severe cytokines and chemotaxis, but lower titers of SARS‐CoV‐2‐specific IgG antibody levels and weaker antiviral functions compared to the young ones.^146^, ^147^ Taken together, although the NHPs have failed to recapitulate the severe diseases such as ARDS (only observed in a few aged African green monkeys) and SIRS, these animals still present the priority as models for studying the immunopathology and evaluating the protective efficacy of vaccines and drugs to combat with SARS‐CoV‐2 infection.^148^, ^149^, ^150^, ^151^, ^152^

## 5 FERRET MODELS

Ferrets (*Mustela putorius furo*) are mammals that have been well used as models to study the infection and transmission of human respiratory viruses, including influenza virus, SARS‐CoV.^153^, ^154^, ^155^ The respiratory tract of ferrets anatomically resembles that of humans, such as the glandular density in the bronchial wall, terminal bronchioles, and proportions of the upper and lower respiratory tract.^156^, ^157^ ACE2 is mainly expressed on glandular and type‐II alveolar epithelial cells of ferrets and shows affinity to the SARS‐CoV infection.^158^, ^159^ Intranasal inoculation of SARS‐CoV‐2 induces the clinical symptoms that observed in COVID‐19 patients, including reduced activity, nasal discharge, sneezing, wheezing, occasional cough, and increased body temperature in infected ferrets.^160^, ^161^ The clinical symptoms are manifested on 2 dpi and recover to the initial state on 12 dpi. The body weight loss is controversial in several studies on SARS‐CoV‐2‐infected ferrets.^162^, ^163^, ^164^ High levels of SARS‐CoV‐2 are detected in the upper respiratory tract, such as nasal turbinate and throat, with peaking between 3 and 4 days and persisting until 8 days before dropping below detection limits on 10 days after inoculation.^161^, ^162^, ^163^ The viral replication is also observed in the trachea, lung, intestine, and brain,^161^, ^164^ suggesting the possibilities of central nervous system injury and oral‐faecal route of transmission. Interestingly, intratracheal inoculation of SARS‐CoV‐2, a route which is reported to cause more severe disease in ferrets when infected with influenza virus^165^, ^166^, ^167^ and in hACE2 mice^25^ when infected with SARS‐CoV‐2, shows a low efficient in establishing an infection with milder diseases in ferrets compared to the route of intranasal inoculation.^164^ The poor outcome may be caused by the relative lower expression of receptor in lower respiratory tract of ferrets for SARS‐CoV‐2 entering. Furthermore, studies on gender and age‐related SARS‐CoV‐2 infection suggest that the aged ferrets, especially the males, suffer more illnesses, such as higher viral loads, longer viral replication, more severe pulmonary disease, and clinical features.^160^, ^168^, ^169^ The skewed distribution captures some features observed in COVID‐19 patients.

In ferrets, SARS‐CoV‐2 infection causes minimal to mild pathologic changes. The nasal cavity develops a mild inflammation with few necrosis of epithelial cells and occasional hemorrhage.^162^, ^167^ Moderate bronchopneumonia occurs as early as 3 dpi, accompanied by infiltration of neutrophils, mononuclear cells, macrophages and lymphocytes into the bronchiolar lumina, and mild necrosis of the bronchiolar epithelial cells.^162^, ^167^ The bronchopneumonia only affects no more than 15% of the lung section on 3 dpi and decreases to less than 5% on 7 dpi.^162^ Thickness of the interalveolar septa and alveolar hyperplasias are observed during SARS‐CoV‐2 infection.^162^, ^164^ Moreover, multifocal hepatitis occurs after inoculation, suggesting the liver damage.^162^

The ferrets are suitable to study the transmission of SARS‐CoV‐2.^170^ Efficient viral transmission between ferrets agrees with recent SARS‐CoV‐2 outbreaks in mink farms. Direct contact with SARS‐CoV‐2‐infected ferrets can induce the transmission of virus to the naïve ferrets, which occurs as early as 2 dpi.^161^, ^171^, ^172^ Transmission via respiratory droplets and/or aerosols is efficient between ferrets over more than 1 m distance,^173^ but shows a delayed infection for 3–7 days^172^ compared to the direct contact. However, in one study, the respiratory droplet shows a low efficiency with only 25% of infection between naïve ferrets.^171^ The pattern of clinical features, viral replication, pathology, and immune response of the infected naïve ferrets resemble those of inoculated ferrets.^161^, ^172^ To date, the ferrets are well used as models to measure the countermeasures against SARS‐CoV‐2 transmission. For example, treatment with a lipopeptide fusion inhibitor, which blocks membrane fusion between the virus and host cell, completely prevents the direct contact of transmission of SARS‐CoV‐2 between ferrets,^174^ suggesting the potential to reduce community transmission of SARS‐CoV‐2. Besides, MK‐4482/EIDD‐2801, a ribonucleoside analog inhibitor, also mitigates SARS‐CoV‐2 infection and blocks transmission in the ferret model.^175^

Involved immune responses include activated T cells, macrophages, and Type I IFN signaling.^160^, ^168^, ^176^ The adaptive response is induced and neutralizing antibodies are produced as early as 8 dpi in ferrets.^162^ The induced immune response protects ferrets from adverse effects when rechallenged the SARS‐CoV‐2, including lower viral replication, no remarkable lesion in the upper and lower respiratory tracts, and no significant bronchopneumonia.^162^, ^163^, ^171^ However, lack of ferret‐specific immunological reagents severely impedes the further investigation of immunological mechanisms in this animal model. Some new technologies such as single‐cell RNA (scRNA) sequencing,^176^ have been employed to resolve this dilemma. The ferret model has been used in the preclinical evaluation of vaccines^177^, ^178^ and antiviral drugs.^179^

## 6 OTHER SPECIES

### 6.1 Cats

Several studies have demonstrated the transmission from human to domestic cats (*Felis catus*).^180^, ^181^, ^182^, ^183^, ^184^ Cats are highly susceptible to airborne transmission and SARS‐CoV‐2 infection, and those who infected by their human owners could transmit the virus to the naïve cats.^185^, ^186^ However, the efficiency of transmission from infected cats to the naïve ones could be attenuated by serial passaging of the virus between cats,^187^, ^188^ and reinfected cats also show no transmissibility.^189^ Intranasal inoculation of SARS‐CoV‐2 induces the viral replication in upper and lower respiratory tract with peaking on 3 dpi and persisting until 5–7 dpi before dropping below detection limits on 14 dpi.^185^, ^188^, ^190^, ^191^ The viral replication is also observed in the spleen, lymph node, liver, heart, olfactory bulb, and gastrointestinal tract.^189^, ^191^ None of clinical feature of disease is found during the infectious period in the current studies, except for one reported loss of body weight,^188^ suggesting the minimal to mild disease in infected cats. Pathological analysis shows that inflammation in nasal turbinate and trachea and moderate interstitial pneumonia accompanied by diffuse alveolar damage occur during the SARS‐CoV‐2 infection.^184^, ^188^, ^189^, ^190^, ^191^ Characteristic changes in lung tissue include swelling and degeneration of bronchiolar epithelial cell, cellular debris, thickened alveolar septa, collagen deposition, mild pulmonary edema, mild‐to‐moderate bronchiolitis with infiltration of monocytes, neutrophils and lymphocytes which are confined predominantly to perivascular sites. The thrombosis is not the common feature in the infected cats.^184^ In addition, SARS‐CoV‐2 infection also causes the gastrointestinal tract injury, such as multifocal inflammation with infiltration of neutrophils and lymphocytes in the intestinal submucosa and muscularis.^188^ The viral antigen expression is observed mainly in epithelial cells of the alveolar and nasal turbinate, enterocytes of the small intestine and submucosal gland of the trachea.^188^, ^189^ The adaptive response is induced and neutralizing antibodies are produced as early as 7 dpi and maintained or increased in titer even on 28 dpi in cats.^190^ Rechallenged cats exhibit faster and more robust humoral immunity against the viral infection.^189^ Although cats may be a potential candidate for studying the asymptomatic to moderate COVID‐19, we should take care of the feasibilities of cats as models for demonstrating the respiratory disease of humans. Additional studies on transmission efficiency need to be further explored, especially cat as an intermediate host between SARS‐CoV‐2 and humans, inflammation and how this mirrors human disease.

### 6.2 Mink

The mink (*Neovison vison*) are mammals that present highly susceptive to the infection of SARS‐CoV^192^ and SARS‐CoV‐2.^193^ The first outbreak of SARS‐CoV‐2 in the mink farm occurs in the Netherlands,^194^ and then infections are reported in more countries.^193^, ^195^, ^196^, ^197^ Studies have demonstrated the high transmission among mink through direct contact or airborne, accompanied by adapted mutations.^198^, ^199^, ^200^, ^201^, ^202^ Notably, the transmission can cause the “back to human” infection and subsequently leads to the prevalence among humans.^198^, ^202^, ^203^ Therefore, measures of prevention and control should be tailored to avoid the potential large‐scale community transmission, regarding the impact of mutants on viral fitness pathogenicity and contagiousness between mink and humans. Intranasal inoculation of SARS‐CoV‐2 induces robust replication of virus in upper and lower respiratory tract, including nasal turbinate, tonsil, soft palate, and trachea, and in lung tissue with peaking on 2 dpi and persisting until 8 dpi before dropping below detection limits on 14 dpi.^204^ The viral replication is also observed in gastrointestinal tract, but not in other organs. Infected mink show mild to severe clinical features, such as decreased feed intake and a maximum 20% of body weight loss.^204^ Pathological analysis shows that inflammation in nasal turbinate is characterized by infiltration of neutrophils, epithelial degeneration, and necrosis.^194^, ^204^, ^205^ Mucus is the main character that enhances the transmission and causes the olfactory function impairment.^206^ Viral infection results in the severe diffuse interstitial pneumonia as well. Characteristic changes in lung tissue include swelling and degeneration of bronchial epithelial cells, thickening of alveolar septa, cellular debris, collagen deposition, hemorrhages, pulmonary edema, pneumocyte proliferation, consolidation and multifocal to diffuse alveolar damage with infiltration of macrophages, monocytes and neutrophils throughout the lungs, and lymphocytes around the vessels.^194^, ^204^, ^205^ The viral infection can cause lethal disease in mink with no gender‐related skewed distribution; however, the naïve mink transmitted by the inoculated ones show alleviated clinical features.^194^, ^204^ Nevertheless, there is scarcely any study on mink as models to investigate the pathogenesis, therapies, and vaccines evaluation.

### 6.3 Fruit bats

The bats are mammals that have been shown as natural reservoirs for a variety of RNA viruses, including SARS‐CoV and MERS‐CoV.^207^, ^208^, ^209^, ^210^, ^211^ These viruses have caused serious transmission between animals and humans and led to the lethal disease. The currently emerged SARS‐CoV‐2 among humans is closely related to β‐coronaviruses which is found in bats,^212^, ^213^ suggesting that SARS‐CoV‐2 might originate in bats. Intranasally inoculated fruit bats (*Rousettus aegyptiacus*) with SARS‐CoV‐2 induces viral replication in upper and lower respiratory tract including nasal epithelium, trachea, and in lung and lung‐associated lymphatic tissue with no clinical feature.^170^ The viral replication in other organs of duodenum, skin, heart, and adrenal gland tissue is at low levels.^170^ However, the transmission can only be found in part of bats at nasal epithelium and trachea. Pathological analysis shows minimal to mild pathological lesions accompanied by cellular debris, edema, and infiltration of neutrophils and lymphocytes, and no pathological change is observed in other organs.^170^ The adaptive response is induced by SARS‐CoV‐2 and neutralizing antibodies are produced as early as 8 dpi in fruit bats.^170^ Although fruit bats are not used in the therapies and vaccines of countermeasures against SARS‐CoV‐2, studies on the bat model may be required for the pathogenesis and transmission between bats and the potential animals else, or humans, and the evolution.

### 6.4 Chinese tree shrews

The Chinese tree shrews (*Tupaia belangeri chinensis*) are mammals that are genetically closer to humans than to rodents, and have been used as models for viral infections, including hepatitis viruses (e.g., HBV and HCV),^214^, ^215^, ^216^ arboviruses (Zika virus and dengue virus)^217^, ^218^ and respiratory viruses (e.g., influenza viruses and human adenovirus B),^219^, ^220^ and so forth. The receptor ACE2 gives an overall homology of 85.47% compared with that of humans and shows affinity to the SARS‐CoV‐2, and is highly expressed in the organs of kidney, lung, liver, spleen, and spinal marrow.^221^ The TMPRSS2and type II transmembrane serine protease 4 (TMPRSS4), two receptors for SARS‐CoV‐2 infection, are also expressed in lung, liver, esophagus, intestine, and kidney.^221^ Combined oral, intranasal, and ocular inoculation of SARS‐CoV‐2 induces viral replication in lung tissues, especially in pneumocytes of young tree shrews (1 year old) with peaking on 3 dpi and gradually dropping below detection limits on 14 dpi; the aged tree shrews (5 to 6 years old) are less susceptible to SARS‐CoV‐2 infection compared to the young ones and show a longer duration of virus shedding in lung tissues with peaking on 7 dpi.^222^ Interestingly, the male and aged tree shrews have more efficiency in virus shedding than the females.^223^ Viral loads in extrapulmonary organs and sera are low or below detection limits.^222^, ^223^ No clinical features are observed expect the increased body temperature particularly in female animals.^223^ Pathological analysis shows the thickened alveolar septa and interstitial hemorrhage in infected tree shrews, accompanied by mild inflammatory infiltrates.^222^, ^223^ However, some observations such as the injury of extrapulmonary organs (brain, heart, liver, kidney, and spleen) are inconsistent, perhaps because of the difference in inoculation route and challenge stocks.^222^, ^223^ Although there is scarcely any study on tree shrews as models to investigate the pathogenesis, therapies, and vaccines evaluation against SARS‐CoV‐2 infection because of their disability to recapitulate the most features observed in COVID‐19 patients, more efforts on tree shrews need to be made to demonstrate the potential intermediate host between animals or humans.

### 6.5 Dogs, pigs, chickens, and ducks

Several studies have shown that animal models including dogs (*Canis lupus familiaris*),^224^ pigs (*Sus scrofa domesticus*),^170^, ^224^ chickens (*Gallus gallus domesticus*),^170^, ^224^, ^225^, ^226^ and ducks (*Anatinae*),^226^ are not susceptible to the SARS‐CoV‐2 infection, despite of *in silico* dock of affinities to SARS‐CoV‐2 for pigs, dogs,^227^, ^228^ and chickens.^229^ Inoculation of SARS‐CoV‐2 induces no clinical features or pathologic changes in these animal models, and the viral replication in chickens, ducks, or pigs which has been reported the infection with SARS‐CoV,^230^ is not observed. Therefore, these animal models may be not suitable for investigating the pathogenesis, therapies, and vaccines evaluation against SARS‐CoV‐2.

## 7 CONCLUSIONS

There are a variety of animal models that have been developed to demonstrate the transmission, pathogenesis, and immunology induced by SARS‐CoV‐2, and to evaluate the immunomodulatory and antiviral drugs and vaccines against COVID‐19. The most important criterion for animal models is to well recapitulate the features that observed in COVID‐19 patients. Genetically modified mice that express hACE2, especially the K18‐hACE2 and CAG‐hACE2 transgenic mice which characterize the severe disease of COVID‐19 patients with ARDS or SIRS, and Syrian hamsters are widely used as rapid models. These animal models are easy to obtain, and have good operability and reproductivity. However, some limitations must be highlighted, such as the different expression of hACE2 among organs in hACE2 transgenic mice, ectopically expressed hACE2 via adenoviral/adenoviral‐associated vectors that may change the tissue or cellular tropism of the virus, the occurrence of key mutations in immunogen such as RBD domain that may lead to the noneffective neutralizations for the mouse‐adapted virus, and the minimal disease in infected naive hamsters. Moreover, NHPs have similar phylogeny and immunology to humans and are widely used for evaluating the protective efficacy of vaccines and drugs; the receptors for SRAS‐CoV‐2 shows different expression in upper and lower respiratory tract, which affects the viral transmission and infection. The association between the expression manner of ACE2 receptors and disease phenotypes need to be considered. Further studies should be required to standardize the challenge stocks, route, and method of viral inoculation, which may lead to the various of diversities in clinical feature, pathology, immunology, and caused mortality.

Understanding the transmission of SARS‐CoV‐2 is vitally important for control of the epidemic disease. At present, Syrian hamsters and ferrets are well characterized as models to study the transmission prevention of SARS‐CoV‐2, because of the comparability of transmission to humans. The cats, mink, fruit bats, and tree shrews are also susceptive to the SARS‐CoV‐2 infection. Moreover, the emerged variants might impart cross‐species transmission of SARS‐CoV‐2 to naïve mice by enhancing receptor binding. Therefore, the potential implications in SARS‐CoV‐2 (naïveté and variants) transmission, such as animal‐to‐animal or animal‐to‐human, and the evolution should be warranted.

To data, all reported animal models cannot completely recapitulate the characters, especially the immunology changes in COVID‐19 patients. It may be beneficial for investigators to apply appropriate options of animal models depending on the scientific goals. Although several models such as mouse and hamster, show age and gender‐related skewed distribution when infected with SARS‐CoV‐2, other risk factors of chronic disease‐related models that correlate to the severe COVID‐19 disease and SARS‐CoV‐2‐induced mortality, including hypertension, diabetes, obesity, and cardiovascular disease, should be further tailored to explore the advanced pathogenesis and the prevention against SARS‐COV‐2. Taken together, animal models provide a fundamental platform to investigate the transmission, pathogenesis, and countermeasures against SARS‐CoV‐2.

## CONFLICTS OF INTEREST

All authors declare no competing interests.

## AUTHOR CONTRIBUTIONS

Z.B., S.L. and X.P. conceived the manuscript. Z.B. collected references and wrote the manuscript. H.Q. and J.Y. designed the figures. S.L. and X.P. revised the manuscript. All authors commented on the manuscript.

## ETHICS APPROVAL

Not applicable.

## Data Availability

Not applicable.
